# Efficacy and safety of fluoroquinolone-containing regimens in treating pulmonary *Mycobacterium avium* complex disease: A propensity score analysis

**DOI:** 10.1371/journal.pone.0235797

**Published:** 2020-07-09

**Authors:** Hisayuki Shuto, Kosaku Komiya, Akihiko Goto, Takamasa Kan, Kokoro Honjo, Sonoe Uchida, Shuichi Takikawa, Tetsuyuki Yoshimatsu, Mari Yamasue, Kazufumi Hiramatsu, Jun-ichi Kadota

**Affiliations:** 1 Internal Medicine, National Hospital Organization Nishi-Beppu Hospital, Beppu, Oita, Japan; 2 Respiratory Medicine and Infectious Diseases, Oita University Faculty of Medicine, Yufu, Oita, Japan; Rutgers Biomedical and Health Sciences, UNITED STATES

## Abstract

**Background:**

Although combination therapy using clarithromycin, rifampicin, and ethambutol is recommended for patients with pulmonary *Mycobacterium avium* complex (MAC) disease, some patients do not tolerate it because of adverse effects or underlying diseases. The efficacy and safety of fluoroquinolone-containing combination regimens as an alternative remain uncertain. This study aimed to compare the efficacy and safety of fluoroquinolone-containing regimens with those of the standard regimens for treating pulmonary MAC disease.

**Methods:**

We retrospectively included consecutive MAC patients who were treated in our hospital between January 2011 and May 2019. Patients treated with fluoroquinolone-containing regimens who had relapsed after treatment with standard regimens were excluded. A propensity score analysis was conducted to reduce selection bias, and the proportions of clinical improvement, defined by chest imaging findings and sputum conversion, were compared between the fluoroquinolone-containing regimen and standard regimen groups.

**Results:**

We analyzed 28 patients who received fluoroquinolone-containing regimens and 46 who received the standard regimen. Fluoroquinolone-containing regimens were more likely selected for patients with cavitary lesions, diabetes mellitus, culture negativity, a low daily physical activity level, a decreased lymphocyte count and an increased CRP level. The propensity score was calculated using these variables (C-statistic of the area under the receiver operating characteristic curve of the propensity score: 0.807, p < 0.0001). The fluoroquinolone-containing regimens were significantly inferior to the standard regimen in clinical improvements (p = 0.002, Log-rank test) in the univariate analysis, but the significance was lost after adjusting for the propensity score (HR 0.553, 95% CI 0.285–1.074, p = 0.080). Six (21%) patients in the fluoroquinolone-containing regimen group and ten (22%) patients in the standard regimen group experienced low-grade adverse effects.

**Conclusions:**

There was no significant difference in clinical improvement between these regimens after propensity score adjustment. A large-scale prospective study is required to validate these results.

## Introduction

*Mycobacterium avium* complex (MAC) is the most commonly isolated pathogen responsible for pulmonary nontuberculous mycobacterium (NTM) disease, particularly in North America and East Asia, and its worldwide prevalence has been gradually increasing since the late 1990s [1, 2]. The American Thoracic Society and Infectious Disease Society of America (ATS/IDSA) guidelines recommend the use of multiple drug regimens that include clarithromycin (CAM) or azithromycin, rifampicin (RFP), and ethambutol (EB) as standard therapy for pulmonary MAC disease [3]. However, sputum culture conversion only occurs in 50%–70% of patients after treatment [4–7].

The guidelines suggest intravenous aminoglycoside administration or surgical resection for the additional treatment of refractory cases or relapse [3]. Previous studies focusing on the efficacy of fluoroquinolones have targeted its use as an additional drug for treating cases [8–10]. However, even in non-refractory cases, other treatment options are required [11] when drug-related adverse events occur, such as liver injury, gastrointestinal disturbance, rash, or optic neuritis [3]. Although fluoroquinolones are shown to have antibacterial activity against MAC *in vivo* and *in vitro* [12–14], there is no clear evidence that they can be used as an alternative in clinical practice. While some studies have shown the non-inferiority of fluoroquinolones to the standard regimens for MAC patients [15, 16], the sample size was small or old quinolones were used. Consequently, it remains uncertain whether new fluoroquinolone-containing regimens are effective for MAC patients in recent clinical settings. Therefore, this study aimed to assess the efficacy and safety of a fluoroquinolone-containing regimen as an alternative rather than as an additional drug compared to the standard regimen in treating MAC disease.

## Methods

### Patients

This retrospective cohort study was conducted at the National Hospital Organization Nishi-Beppu Hospital in Oita Prefecture, Japan. Patients with pulmonary MAC diagnosed by ATS/IADS microbiological criteria on hospital admission between January 2011 and May 2019 were enrolled [3]. Patients treated with a combination of CAM, EB, and RFP were assigned to the standard regimen group. Patients treated with a modified regimen that included a fluoroquinolone substitution instead of RFP and/or EB due to occurrence or concerns regarding drug-related adverse effects were assigned to the fluoroquinolone-containing regimen group. Patients with an NTM species isolated from the sputum other than MAC were excluded. Patients who were treated with fluoroquinolone-containing regimens or standard regimens for less than four weeks, or who were treated with a single- or two-drug regimen not including a fluoroquinolone, were also excluded. Moreover, we excluded patients in whom a fluoroquinolone was used in addition to standard therapy for refractory cases such as those with CAM-resistant strains. We did not exclude fluoroquinolone-resistant strains because the role of the minimum inhibitory concentrations (MICs) of fluoroquinolones for MAC has not been fully elucidated [17].

The estimated sample size was calculated using G*Power (2-tailed, α error = 0.05, power = 0.8, effect size = 0.3), and a total sample size of 88 patients including 44 patients treated with fluoroquinolone-containing regimens and 44 patients with standard regimens was deemed necessary according to previous studies [16]. The study protocol was approved by the Institutional Ethics Committee (Approval Number 1–1; Approval Date July 29, 2019). The need for informed consent was waived because of the retrospective nature of the study.

### Data collection

The following patient data were obtained from the medical records: age, gender, body mass index, daily physical activity level on admission, underlying diseases, laboratory data, presence of respiratory failure, treatment regimen, and drug-related adverse effects. The MICs of CAM and LVFX were routinely tested in the hospital because the MIC of CAM is known to be associated with prognosis, and fluoroquinolones are occasionally used for MAC treatment [18]. The results of the susceptibility testing of the other drugs such as RFP or EB for MAC were not documented because they are not considered correlated with clinical efficacies. In addition to the information of species, smear, and culture positivity at the time of treatment initiation, the MICs of CAM and LVFX were collected. The daily physical activity on admission was graded using the Eastern Cooperative Oncology Group Performance Status scale [19] as follows: 0 –fully active, able to perform all pre-disease activities without restrictions; 1 –restricted in physically strenuous activity but ambulatory and able to perform light and sedentary work; 2 –ambulatory and capable of all self-care but unable to perform any work activities, up and about more than 50% of waking hours; 3 –capable of only limited self-care, confined to a bed or a chair more than 50% of waking hours; and 4 –completely disabled, cannot perform any self-care, completely confined to a bed or a chair.

Drug-related adverse effects were elucidated according to the Common Terminology for Criteria for Adverse Events, version 5.0. Two respiratory medicine physicians independently evaluated cavitary lesions, bronchiectasis, and areas of lung disease on plain radiography and high-resolution computed tomography (HRCT) imaging of the chest.

### Outcomes and definitions

The primary outcome was clinical improvement with antimicrobial treatment. Improvement was defined as either sputum culture conversion (three consecutive negative smears or two consecutive negative cultures) or improvement in chest radiographic imaging findings. CAM resistance was defined by an MIC ≥32 μg/mL.

### Statistical analysis

Statistical analyses were performed using IBM SPSS version 24 software (IBM, Armonk, NY, USA). P < 0.05 was considered statistically significant. The kappa statistic was used to assess the concordance of imaging evaluations. Propensity scores were used to reduce selection bias for treatment with fluoroquinolone-containing regimes and were estimated based on variables that differed between the fluoroquinolone-containing regimen group and the standard regimen group for 0.8 > area under the curve. Kaplan-Meier curves were constructed using the log-rank tests to compare the time to achieve the primary outcome between treatment groups. Multivariate analyses were conducted using the Cox proportion hazards model after adjustment for propensity scores to reduce selection bias between treatment groups.

## Results

### Patient characteristics

**Fig 1:**
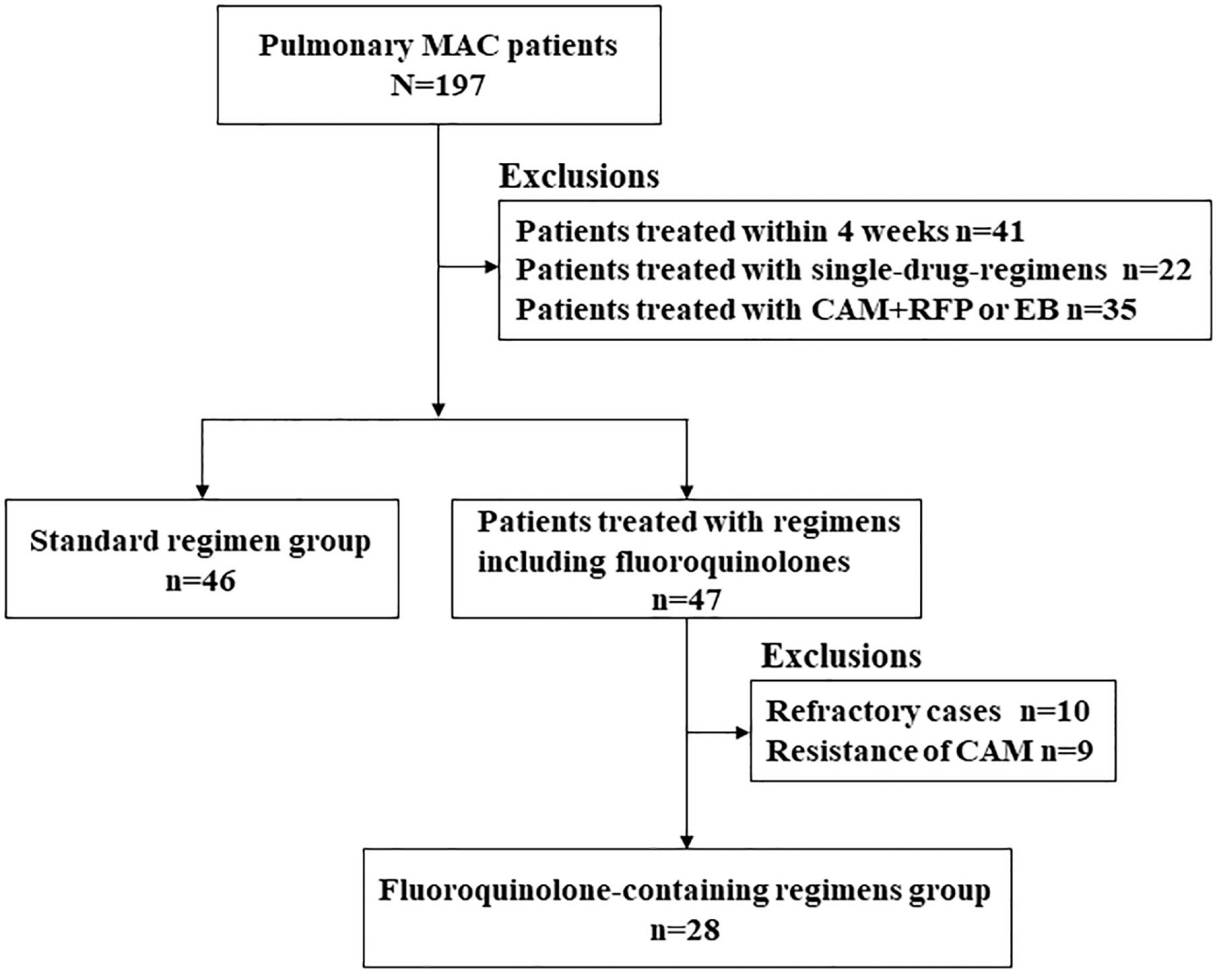
Flowchart of participant selection.

A total of 197 patients met the ATS/IDSA microbiological diagnostic criteria for pulmonary MAC. Of these, 46 patients were treated with a standard regimen and 47 were treated with a fluoroquinolone-containing regimen. All 46 patients in the standard regimen group were treated with the same regimen: a combination of RFP, EB and CAM, and were included for analysis. After excluding 10 refractory patients and 9 patients with CAM resistance, 28 patients were included in the fluoroquinolone-containing regimen group for analysis (Fig 1).

Table 1 shows the specific fluoroquinolone-containing regimens included in this study. All patients in the fluoroquinolone-containing regimen group were treated with combinations that included CAM. No patients were treated with moxifloxacin (MFLX) or gatifloxacin (GFLX) in this study. Drug susceptibility tests for CAM did not have to be repeated in any patients. Fluoroquinolone-containing regimens were generally selected for patients with cavitary lesions, diabetes mellitus, negative culture, low daily physical activity levels, lower lymphocyte counts, and higher C-reactive protein level (Table 2). Propensity scores were calculated based on these variables, and fluoroquinolone-containing regimens were shown to have a good C-statistic (0.807, 95% confidence interval (CI) 0.708–0.905; p < 0.0001).

**Table 1:** The details of fluoroquinolone-containing regimens.

regimens	n
CAM+RFP+LVFX	12
CAM+RFP+STFX	2
CAM+EB+LVFX	3
CAM+EB+STFX	2
CAM+EB+STFX+SM	1
CAM+LVFX	5
CAM+STFX	2
CAM+STFX+SM	1
Total	28

CAM: clarithromycin, RFP: rifampicin, EB: ethambutol

LVFX: levofloxacin, STFX: sitafloxacin, SM: streptomycin

**Table 2:** Baseline characteristics with crude odds ratio for fluoroquinolone-containing regimens.

	FQ-containing regimens (n = 28)	Standard regimen (n = 46)	Odds ratio	p value
Gender (female)	19 (68)	33 (72)	0.832 (0.300–2.307)	0.723
Age (years)	73 (65–78)	71 (62–79)	1.022 (0.978–1.068)	0.339
BMI (kg/m2)	18.6 (16.4–21.6)	18.5 (16.3–20.2)	1.031 (0.868–1.224)	0.728
PS (≥1)	26 (93)	32 (70)	5.688 (1.184–27.321)	0.030
Current smoker	5 (19)	9 (20)	0.934 (0.278–3.146)	0.913
COPD	4 (14)	4 (9)	1.750 (0.401–7.641)	0.457
Cardiac diseases	1 (4)	3 (7)	0.531 (0.052–5.368)	0.592
Diabetes mellitus	4 (14)	1 (2)	7.500 (0.793–70.917)	0.079
WBC (10^3^/μl)	5.7 (4.1–8.1)	5.9 (4.6–7.0)	1.128 (0.928–1.371)	0.228
Lymphocytes (10^3^/μl)	1.1 (0.94–1.3)	1.3 (0.98–1.8)	0.335 (0.116–0.968)	0.043
Hb (g/dl)	12.2 (11.3–12.9)	12.5 (11.5–13.7)	0.812 (0.562–1.172)	0.266
PLT (10^4^/μl)	21.7 (18.6–31.1)	25.1 (19.4–29.6)	0.998 (0.950–1.049)	0.945
CRP (mg/dl)	0.55 (0.08–3.25)	0.31 (0.06–0.89)	1.241 (1.001–1.538)	0.049
Alb (g/dl)	3.8 (3.3–4.1)	3.8 (3.3–4.2)	0.924 (0.276–3.087)	0.897
AST (U/l)	22 (17–26)	21 (17–24)	0.979 (0.925–1.037)	0.466
BUN (mg/dl)	15 (12–18)	15 (12–18)	1.024 (0.923–1.136)	0.649
Cr (mg/dl)	0.63 (0.56–0.81)	0.65 (0.54–0.75)	2.460 (0.117–51.68)	0.562
Smear positive	14 (50)	23 (50)	1.000 (0.391–2.559)	1.000
Culture positive	19 (68)	38 (83)	0.444 (0.148–1.335)	0.149
*M*. *avium*	13 (46)	16 (35)	1.625 (0.623–4.240)	0.321
*M*. *intracellulare*	15 (54)	30 (65)	0.615 (0.236–1.606)	0.321
MIC				
CAM	0.25 (0.13–1.00)	0.13 (0.060–0.25)	2.409 (0.794–7.311)	0.121
LVFX	1.00 (1.00–2.00)	1.00 (0.50–2.00)	1.121 (0.691–1.818)	0.644
Cavitary lesion	19 (68)	16 (35)	3.958 (1.458–10.745)	0.007

Data are presented as the number (%) or median (interquartile range).

FQ: fluoroquinolone BMI: body mass index, PS: performance status, COPD: chronic obstructive pulmonary disease, WBC: white blood cell, Hb: hemoglobin, PLT: platelet, CRP: C-reactive protein, Alb: albumin, AST: aspartate transaminase, BUN: blood urea nitrogen, Cr: creatinine, MIC: minimum inhibitory concentration, CAM: clarithromycin, LVFX: levofloxacin

### Efficacy and adverse events

**Fig 2:**
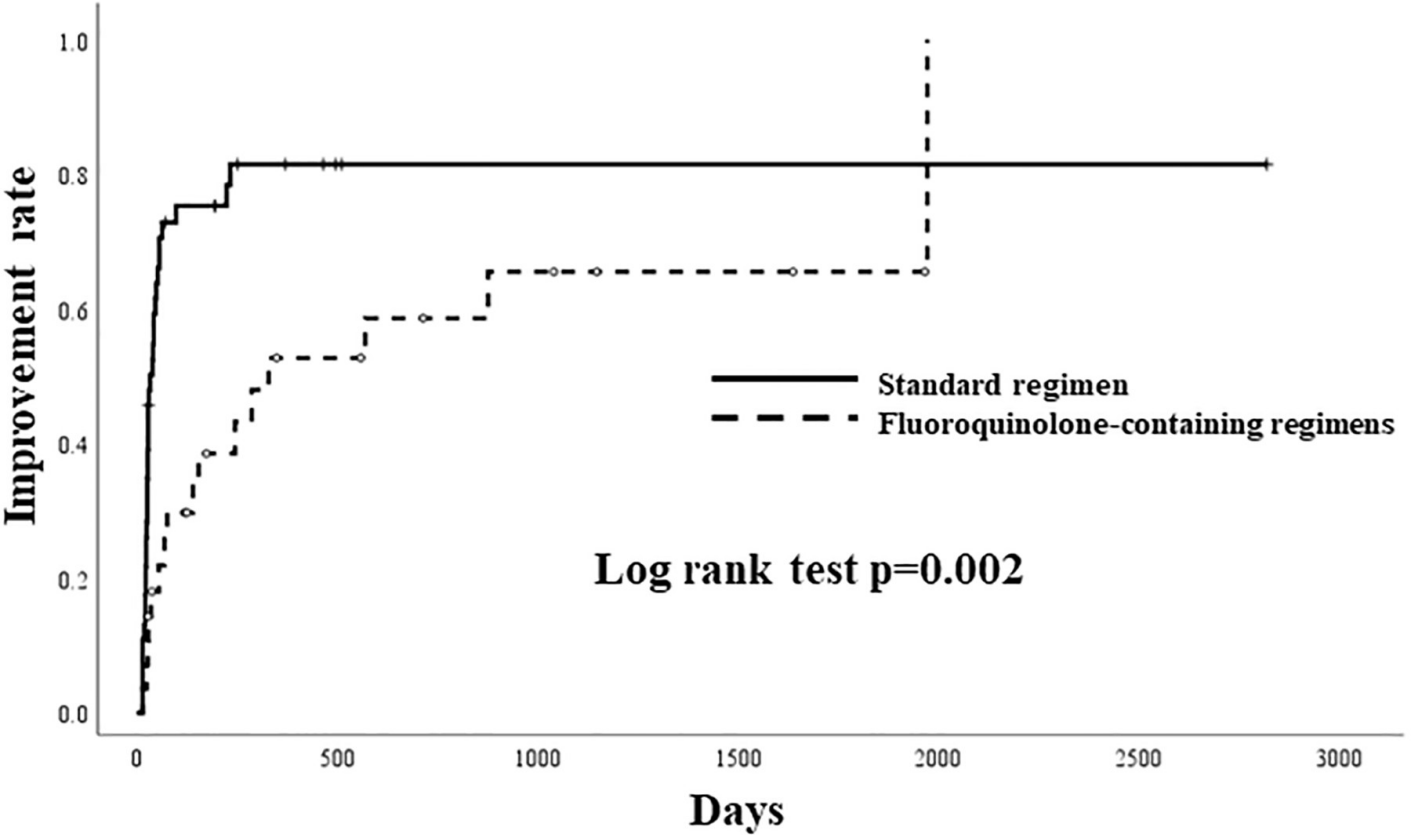
Kaplan-Meier curves showing time to clinical improvement of the fluoroquinolone-containing regimens or standard regimen in patients with pulmonary *Mycobacterium avium* complex disease.

Thirty-six of the 46 patients (78%) in the standard regimen group and 16 of the 28 (57%) patients in the fluoroquinolone-containing regimen group achieved clinical improvement in the univariate analysis (p = 0.003), as shown in Table 3, and the Kaplan-Meier curves constructed using the log-rank test showed a significant advantage of the standard regimen over the fluoroquinolone-containing regimens (p = 0.002, Log-rank test, in Fig 2). However, after adjustment using the propensity scores, the statistical significance disappeared (HR 0.553, 95% CI 0.285–1.074, p = 0.080). The kappa value for judging significant improvements on chest HRCT findings between the two respiratory medicine specialists was 0.745.

**Table 3:** Predictors of improvement in patients with pulmonary *Mycobacterium avium* complex disease.

	Improvement group (n = 52)	Non-improvement group (n = 22)	Hazard ratio	p value
FQ-containing regimens	16 (31)	12 (55)	0.395 (0.215–0.725)	0.003
Gender (female)	38 (73)	14 (64)	1.127 (0.608–2.090)	0.704
Age (years)	72 (66–79)	73 (60–79)	1.020 (0.995–1.046)	0.119
BMI (kg/m2)	18.6 (16.3–20.9)	18.2 (16.6–21.2)	0.981 (0.886–1.086)	0.710
PS (≥1)	39 (75)	19 (86)	0.555 (0.293–1.049)	0.070
Current smoker	8 (16)	6 (27)	0.674 (0.316–1.437)	0.307
COPD	4 (8)	4 (18)	0.484 (0.174–1.345)	0.164
Cardiac diseases	4 (8)	0 (0)	2.031 (0.725–5.690)	0.178
Diabetes mellitus	4 (8)	1 (5)	1.199 (0.430–3.343)	0.729
WBC (10^3^/μl)	5.7 (4.6–7.5)	6.3 (4.3–7.8)	0.989 (0.896–1.091)	0.826
Lymphocytes (10^3^/μl)	1.2 (0.97–1.7)	1.3 (0.82–1.5)	1.427 (0.803–2.536)	0.225
Hb (g/dl)	12.1 (11.3–13.3)	12.5 (11.5–13.2)	1.021 (0.817–1.277)	0.852
PLT (10^4^/μl)	23.4 (19.3–30.0)	23.2 (18.4–29.5)	1.003 (0.975–1.032)	0.817
CRP (mg/dl)	0.34 (0.06–1.88)	0.47 (0.07–1.28)	1.010 (0.918–1.112)	0.831
Alb (g/dl)	3.7 (3.3–4.2)	3.9 (3.4–4.1)	0.983 (0.480–2.013)	0.962
AST (U/l)	21 (17–25)	21 (18–26)	0.998 (0.968–1.029)	0.893
BUN (mg/dl)	15 (13–18)	14 (10–16)	1.004 (0.948–1.063)	0.889
Cr (mg/dl)	0.65 (0.56–0.74)	0.64 (0.53–0.85)	0.516 (0.083–3.196)	0.477
Smear positive	27 (52)	10 (46)	1.134 (0.657–1.958)	0.652
Culture positive	47 (90)	10 (46)	5.929 (2.335–15.051)	<0.001
*M*. *avium*	21 (40)	8 (36)	1.163 (0.666–2.032)	0.596
*M*. *intracellulare*	31 (60)	14 (64)	0.860 (0.492–1.502)	0.596
MIC				
CAM	0.13 (0.060–0.50)	0.13 (0.13–0.50)	0.837 (0.610–1.148)	0.270
LVFX	1.00 (0.50–1.00)	1.50 (1.00–2.00)	0.793 (0.569–1.104)	0.169
Cavitary lesion	22 (42)	13 (59)	0.635 (0.365–1.104)	0.107

Data are presented as the number (%) or median (interquartile range).

FQ: fluoroquinolone BMI: body mass index, PS: performance status, COPD: chronic obstructive pulmonary disease, WBC: white blood cell, Hb: hemoglobin, PLT: platelet, CRP: C-reactive protein, Alb: albumin, AST: aspartate transaminase, BUN: blood urea nitrogen, Cr: creatinine, MIC: minimum inhibitory concentration, CAM: clarithromycin, LVFX: levofloxacin

Six patients (21%) in the fluoroquinolone-containing regimen group experienced adverse effects. Anorexia (grade 1), nausea (grade 1), myalgia (grade 2), and hypoglycemia (grade 1) occurred in one patient each, and gastric ulcers (grade 2) were observed in two patients. Two patients in the fluoroquinolone-containing regimen group discontinued treatment even though they only experienced low-grade adverse effects.

Ten patients (22%) in the standard regimen group experienced adverse effects. Gastralgia (grade 1) was observed in four patients, anorexia (grade 1) in two patients, nausea (grade 1) in two patients, rush (grade 1) in two patients, and dystopia (grade 1) in one patient. One patient in the standard regimen group discontinued treatment.

## Discussion

This study demonstrated no significant difference in clinical improvement between the standard and fluoroquinolone-containing regimens for the treatment of non-refractory pulmonary MAC disease. Although the univariate analysis showed that the standard regimen was superior, after propensity score adjustment in the multivariate analysis, the statistical significance disappeared.

In previous studies, ciprofloxacin (CPFX) and GFLX were used as alternatives for CAM in combination with EB and RFP and were found to be equivalent in efficacy to the standard regimen [15, 16]. However, in our univariate analysis, the standard regimen had a significant advantage over fluoroquinolone-containing regimens. Four possible reasons must be considered to explain this efficacy gap. First, the fluoroquinolone-containing regimens varied between studies with respect to the specific fluoroquinolone utilized. LVFX and sitafloxacin (STFX) were used more frequently in our study; some previous studies have shown that STFX, MFLX, and GFLX have better antibacterial activity against MAC than LVFX or CPFX *in vitro* [20–22]. Indeed, one study suggested that the combination of CAM and LVFX causes unfavorable clinical outcomes for patients treated for pulmonary MAC disease [23]. However, the results cannot rule out the efficacy of LVFX as an alternative drug because the study does not exclude refractory cases from the sample population. To assess the efficacy or safety of fluoroquinolone-containing regimens for pulmonary MAC disease treatment more precisely, it must be clarified whether the study sample also included refractory cases, such as those having CAM resistance, or non-refractory cases using fluoroquinolones as alternatives for the drug among the standard combination regimen. Second, the MICs were not re-examined once an isolated strain was confirmed as CAM-sensitive. However, CAM resistance is likely to be induced when CAM is used in combination with a fluoroquinolone [24]. In this study, seven patients were treated with this particular combination; thus, the development of CAM resistance might have influenced the outcome. Third, some patients began treatment with a fluoroquinolone-containing regimen after the standard regimen was used for a certain period and then discontinued due to adverse effects. In these patients, the efficacy of the fluoroquinolone-containing regimen might have been underestimated. Finally, CAM can decrease the efficacy of fluoroquinolones [20]. One study of mice with pulmonary MAC disease showed that CAM in combination with a fluoroquinolone had a lower efficacy than CAM alone [14]. However, there is no clear evidence regarding which regimen is more effective in humans.

The main strength of our study was the direct comparison of fluoroquinolone-containing regimens as an alternative to the standard regimen recommended by ATS/IDSA guidelines; additionally, we statistically adjusted real world clinical data using the propensity score. However, some limitations should be noted. First, the interval from treatment initiation to the evaluation of efficacy varied depending on the treating physician, which may have introduced measurement bias. Second, there is no objective method for determining improvement on plain chest radiography. We defined clinical improvement as either sputum conversion or the improvement of chest radiography imaging because some patients had a negative sputum smear or culture at the beginning of treatment. Several previous studies defined sputum conversion at 12 months as clinical improvement [4–7], but it remains uncertain whether sputum conversion or improvement of chest radiography affects long-term prognosis. Third, our sample did not include enough patients to reach a power of 0.80. The power based on the actual number of included patients was 0.73, which was slightly lower than expected. Unfortunately, we could not extend the inclusion period (i.e., before January 2011) because of difficulties extracting the data from non-digital medical records. Fourth, the regimens in the fluoroquinolone-containing group varied, as shown in Table 1. No patients were treated with MFLX or GFLX, likely because MFLX is not covered for MAC by universal insurance in Japan and GFLX has not been available since 2008. Further studies based on these data with standardization of the fluoroquinolone regimen and combination drugs are required. Finally, there are still some biases derived from the retrospective nature of this study, although the selection bias regarding which regimen was applied was adjusted by the propensity scores. For example, in this study, the fluoroquinolone-containing regimen was significantly more frequently selected for patients with cavitary lesions. Disease type, such as fibrocavitary disease or nodular bronchiectatic disease in MAC, is known to be associated with disease prognosis [3]. We adjusted for this factor using propensity scores to reduce selection bias, but another weakness of this analysis was the exclusion of other unmeasured confounders. Randomized controlled trials are ideal for reaching definitive conclusions. Furthermore, the MICs for CAM and LVFX were identical between the fluoroquinolone-containing regime group and the standard regime group, but these values may be increased after antibiotic treatment is initiated. To assess the efficacy of treatment with these regimens correctly, the MICs must be re-examined at regular intervals, and the correlation between the values and clinical responses must be evaluated as mentioned previously.

In conclusion, our study showed the possibility of no significant difference in clinical improvement between the standard regimen and fluoroquinolone-containing regimens as an alternative for the treatment of pulmonary MAC after adjustment by propensity scores. Although the incidence of adverse events among patients treated with a fluoroquinolone-containing regimen seems tolerable compared with those in patients treated with the standard regimen [25], the number of patients who discontinued a fluoroquinolone-containing regimen in this study was not negligible. A future prospective study with a more objective outcome measure that uses a specific fluoroquinolone (e.g., STFX), regular monitoring of the CAM MIC during treatment, and a standardized follow-up period is necessary to validate the efficacy and adverse effects of fluoroquinolone-containing regimens and to identify prognostic factors.

## Supporting information

S1 Data (XLSX)

## Abbreviations

ATS/IDSA: The American Thoracic Society and Infectious Disease Society of America
BMI: body mass index
CAM: clarithromycin
CPFX: ciprofloxacin
CTCAE: Common Terminology for Criteria for Adverse Events
EB: ethambutol
GFLX: gatifloxacin
HRCT: high-resolution computed tomography
LVFX: levofloxacin
MAC: *Mycobacterium avium* complex
MFLX: moxifloxacin
MIC: minimum inhibitory concentration
NTM: nontuberculous mycobacterium
RFP: rifampicin
STFX: sitafloxacin
